# The Scaffold Protein Liprin β-1 (PPFIBP1) and the Intermediate Filament Synemin: Potential New Markers of Lymphatic Endothelial Cells

**DOI:** 10.3390/cells15121064

**Published:** 2026-06-10

**Authors:** Jürgen Becker, Jörg Wilting

**Affiliations:** Department of Anatomy and Cell Biology, University Medical Center Göttingen, Kreuzbergring 36, 37075 Göttingen, Germany; juergen.becker@med.uni-goettingen.de

**Keywords:** liprin β-1, PPFIBP1, synemin, desmin, SPHK1, lymphatic vessel

## Abstract

**Highlights:**

**What are the main findings?**

- Lymphatic endothelial cells exhibit considerable molecular heterogeneity depending on their location within the lymphatic network. PPFIBP1 seems to be a promising marker of lymphatic endothelial cells in initial lymphatics, lymph collectors and lymph nodes at the RNA and protein level.
- Synemin (aka desmuslin) seems to be a promising marker of lymphatic endothelial cells in initial lymphatics especially at the protein level.

**What are the implications of the main findings?**

- The new markers may help to better characterize the lymphatic networks in various organs and during embryonic development.
- The new markers may help to better classify lymphatics in pathological tissues such as vascular malformations and cancers, which may have an influence on therapeutic strategies.

**Abstract:**

There are a few molecules that are regularly used as markers for lymphatic endothelial cells (LECs) such as the adhesion molecule CD31/PEACAM1, the transcription factor PROX1, the Vascular Endothelial Growth Factor Receptor-3 (VEGFR3/*FLT4*), the glycoprotein podoplanin, and the hyaluronan receptor LYVE1. However, none of the molecules are exclusively expressed in LECs, and there is molecular and functional heterogeneity of LECs in initial lymphatics, lymphatic collectors and lymph nodes. Therefore, a combination of markers must be applied to identify lymphatics. This is particularly true for the characterization of conditions such as lymphatic malformations or cancers, in which the molecular profile of vessels may be variable or abnormal. Here we present two molecules that can help distinguish between endothelial cells of blood and lymphatic vessels: the scaffold protein liprin β-1 (PPFIBP1) and the intermediate filament synemin. We collected own data on the RNA and protein expression of the two molecules in humans, and studied publicly available databases. PPFIBP1 appears to be a suitable marker of LECs in initial lymphatics, collectors and lymph nodes, while synemin appears to be more restricted to initial lymphatics. We hope this will stimulate monoclonal antibody development and help expand the range of LEC markers in health and disease.

## 1. Introduction

In recent years, a group of molecules have been identified that can serve as markers for lymphatic endothelial cells (LECs). In humans, probably more obviously than in mice, there is molecular heterogeneity of LECs of initial lymphatics, which take up interstitial fluid and hyaluronan, vs. lymphatic collectors, which actively transport lymph centripetally [1]. Accordingly, lymphatic malformations (LMs) derived from either initial lymphatics or collectors express a heterogenous set of LEC markers. In particular, D2-40 (podoplanin) and the hyaluronan receptor LYVE1 are less sensitive and less frequently expressed in macro-cystic LMs, while CD31 (PECAM1), the transcription factor PROX1, and the Vascular Endothelial Growth Factor Receptor-3 (VEGFR3/*FLT4*) are more consistently found [2,3,4] (https://www.pathologyoutlines.com/topic/softtissuelymphangioma.html; accessed on 22 May 2026). VEGFR3, however, is upregulated in tumor blood vessels [5]. Therefore, additional markers may be helpful for histological characterization of LECs.

Using RNASeq we studied three human foreskin-derived LEC isolates under normoxic and hypoxic conditions [6,7]. The cell isolates were highly pure and were characterized by the expression of the typical markers of initial lymph vessels. We systematically analyzed the RNASeq data and identified two structural molecules that can potentially serve as LEC markers at the RNA and protein levels: the scaffold protein liprin β-1 (PPFIBP1) and the intermediate filament synemin (SYNM).

Liprins are highly conserved scaffold proteins in the cortical cytoplasm that bind diverse target proteins to form large complexes capable of regulating various cellular activities [8]. They belong to the family of leukocyte common antigen-related (LAR) transmembrane tyrosine phosphatase-interacting proteins [9]. They are essential components of cellular microcompartments such as focal adhesions and the presynaptic termini of neurons [10,11]. Liprins form homodimers via the N-terminal coiled-coil region, and heterodimers via their C-terminal sterile α motif (SAM) domains. The liprin family is composed of two subgroups: liprin α1-4 and liprin β1-2 [10]. In vitro, significantly higher expression of liprin β-1 (PPFIA binding protein 1; gene name: *PPFIBP1*) was detected in human intestinal LECs as compared to dermal LECs, and a function for the maintenance of lymphatic vessel integrity was shown in Xenopus tadpoles [12]. No further studies on the expression and function of *PPFIBP1* in human LECs have been conducted to date.

Synemin (*SYNM*; aka desmuslin, *DMN*) is a large (1565 AA) type-VI intermediate filament (IF) that is well known for its expression in all types of muscle cells [13]. It fulfills important physiological roles in the muscle cell cytoskeleton [14]. It forms heteromeric IFs with desmin and/or vimentin. Of particular physical significance are its interactions with cytoskeletal proteins dystrophin, α-dystrobrevin, talin-1, utrophin and vinculin in striated muscle. There, it is able to link these heteromeric IFs to adherens junctions, including costameres, neuromuscular junctions, and myotendinous junctions [13,15,16]. Although dominant in muscle cells, expression of SYNM/DMN in some glial, fibrous and malignant tissues has been observed [17,18,19,20]. Expression in LECs has not been studied yet.

## 2. Materials and Methods

### 2.1. Cell Culture

We studied three well-characterized human dermal lymphatic endothelial cell isolates (HD-LECs). Validated cells were freshly purchased from PromoCell (Heidelberg, Germany). Further characterization and culturing were described before [7]. The cells were tested for common human viruses and were found to be virus-free. Lymphatic malformation-derived LECs were described in detail before [21].

### 2.2. RNA Sequencing

RNASeq of defined lymphatic endothelial cells was performed as described [7].

### 2.3. Immunofluorescence (IF)

IF was performed on human foreskin derived from operations performed at the University Medical Center Goettingen (UMG). Specimens were fixed in 4% paraformaldehyde for 1 h, embedded in tissue freeze medium and sectioned at 10 µm. Studies were performed with the informed consent of the patients or their parents, and were approved by the ethics committee of the UMG (application No. 18/1/18). Primary antibodies were: mouse anti-human CD31 (BD Pharmingen, San Jose, CA, USA, dilution 1:50); rabbit anti-human synemin (Atlas antibodies, Stockholm, Sweden, HPA040066, dilution 1:2000); mouse anti-human desmin (clone D33, Agilent/Dako, Waldbronn, Germany, ready-to-use); mouse anti-human podoplanin (clone D2-40, Agilent/Dako, dilution 1:200); rabbit anti-human PPFIBP1 (Sigma, Taufkirchen, Germany, Atlas antibodies, HPA001924, dilution 1:1000). Secondary antibodies were: Alexa 488-conjugated goat anti-mouse IgG (H + L) (Invitrogen, Thermo Fischer Scientific, Waltham, MA, USA, dilution 1:200), and Alexa 594-conjugated goat anti-rabbit IgG (H + L) (Invitrogen, dilution 1:200). Nuclei were counterstained with Dapi.

### 2.4. Immunohistochemistry

We compared our RNASeq expression data with protein expression by systematically studying the Human Protein Atlas (https://www.proteinatlas.org/). All figures shown here can be found and further studied at variable magnification in this repository [22].

## 3. Results

In recent years, we studied the transcriptome of human LECs, either derived from healthy foreskin [6,7], healthy lymph collectors [1] or from lymphatic malformations [21]. Based on this data, we have attempted to identify markers—beyond those already known—that can aid in the detection of LECs in health and disease, and help distinguish them from BECs. Structural proteins are likely to be the most valuable in this context, as they are subject to little or no fluctuation in short- or medium-term regulatory processes, and are regularly used in disease diagnostics [23]. Here we describe two molecules that appear to fulfill the criteria of a marker: PPFIBP1 (liprin β1) and synemin (*SYNM*; aka desmuslin, *DMN*).

### 3.1. Liprin β1/PPFIBP1

We found expression of *PPFIBP1* in three human foreskin-derived LEC lines in a range comparable to that of the LEC markers *PROX1* and *FLT4*/VEGFR-3. Expression of PPFIBP1 was stable under hypoxia (Table 1).

**Figure 1:**
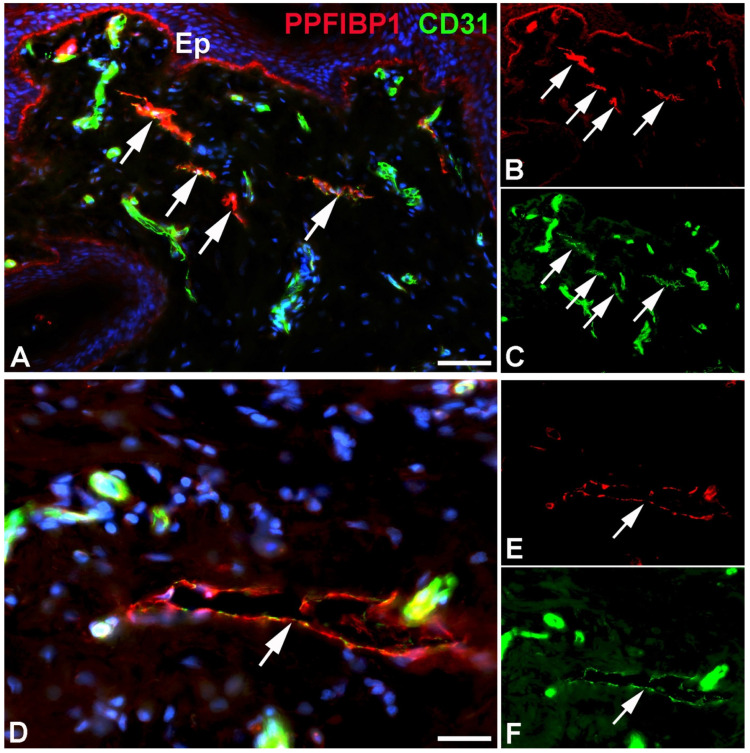
Immunofluorescence studies on PPFIBP1 in human foreskin. Anti-PPFIBP1 (red in (**B**,**E**)) and anti-CD31 (green in (**C**,**F**)) shows expression of PPFIBP1 in CD31-weak lymphatics (arrows). Blood vessels strongly express CD31. (**A**,**D**) Merged images. PPFIBP1 is also found in basal cells of the epidermis (Ep). Nuclei are counterstained with DAPI (blue). Bar = 160 µm in (**A**), and 40 µm in (**D**).

**Figure 2:**
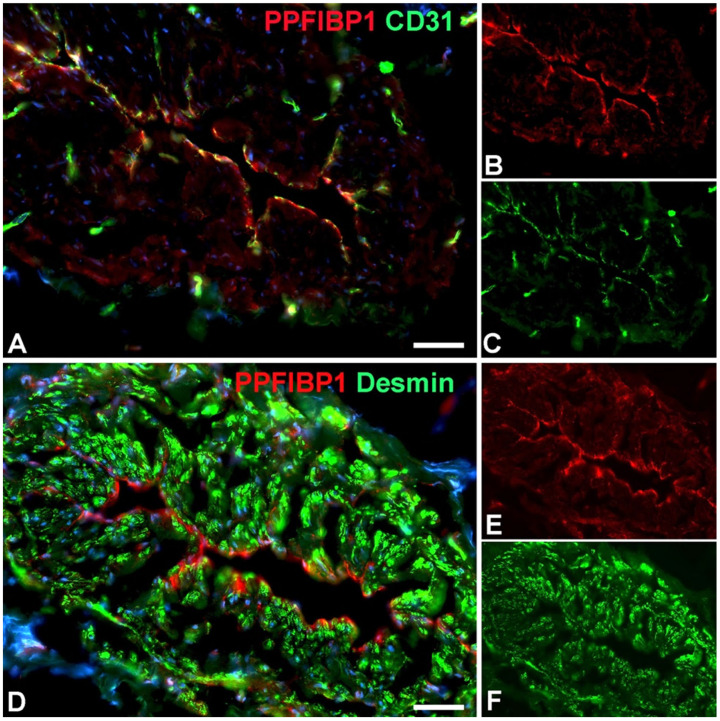
Immunofluorescence studies of PPFIBP1 in human thigh subcutaneous lymph collectors. (**A**–**C**) Anti-PPFIBP1 (red) with anti-CD31 (green), and (**D**–**F**) anti-PPFIBP1 (red) with anti-Desmin (green). Note expression of PPFIBP1 in CD31-weak LECs of the collector. Blood vessels (vasa vasorum) of the collector strongly express CD31. Desmin typically stains smooth muscle cells. (**A**,**D**) Merged images. Nuclei are counterstained with DAPI (blue). Bar = 80 µm in (**A**,**D**).

We studied PPFIBP1 in frozen sections of human foreskin in combination with the endothelial marker CD31/PECAM1. Typically, CD31 is highly expressed on BECs where it marks the cell membrane continuously, while it is found at lower levels and in a focal pattern in LECs. We found robust expression of PPFIBP1 in CD31-weak lymphatics, whereas blood vessels appeared to be constantly PPFIBP1-negative (Figure 1). The basal cells of the epidermis also showed expression of PPFIBP1. Next, we studied lymph collectors from the hypodermis of the thigh isolated for autologous transplantation. These collectors are especially muscle-rich and possess vasa vasorum. Again, PPFIBP1 was found in the CD31-weak LECs, while the vasa vasorum were PPFIBP1-negative (Figure 2A–C). The smooth muscle cells of the media were confirmed with anti-desmin staining (Figure 2D–F).

**Figure 3:**
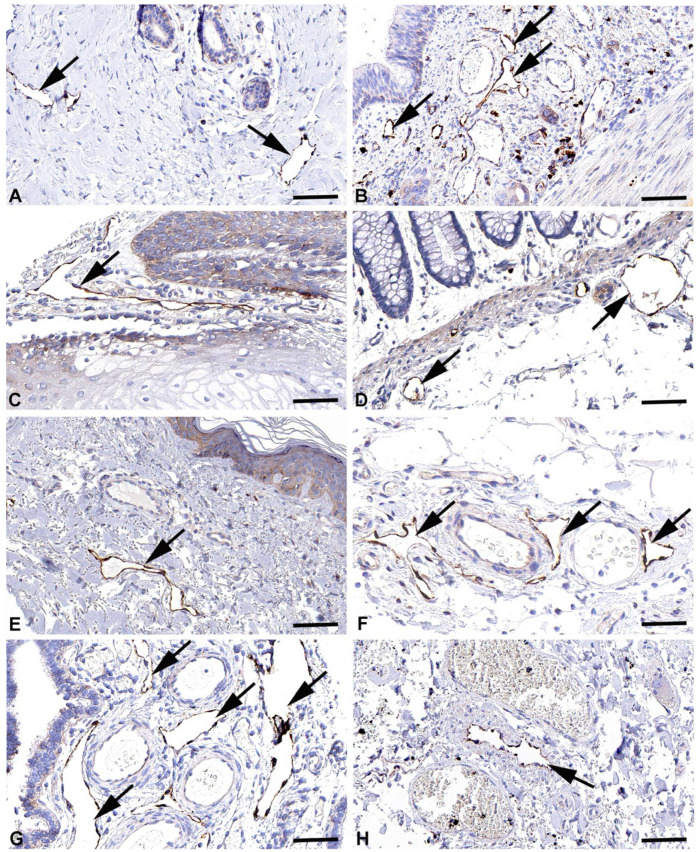
Immunohistochemical studies on PPFIBP1 in adult human tissues. Note the staining of initial lymphatics and lymph collectors (arrows). Data from the Human Protein Atlas [22]. (**A**) Breast, patient id 2773, female 23 y; antibody HPA001924; Bar = 80 µm. (**B**) Bronchus, patient id 2393, female 54 y; antibody HPA001924; Bar = 60 µm. (**C**) Oral tissue, patient id 2406, female 84 y; antibody HPA001924; Bar = 40 µm. (**D**) Rectum, patient id 2060, female 66 y; antibody HPA001924; Bar = 40 µm. (**E**) Skin, patient id 2553, male 71 y; antibody HPA001924; Bar = 40 µm. (**F**) Smooth muscle (arterial) of colon, patient id 2404, male 67 y; antibody HPA001924; Bar = 40 µm. (**G**) Fallopian tube, patient id 1830, female 29 y; antibody HPA001924; Bar = 40 µm. (**H**) Small intestine, lymph collector, patient id 2411, male 71 y; antibody HPA001924; Bar = 80 µm.

Next, we systematically studied immunohistochemical tissues of the Human Protein Atlas [22] and observed PPFIBP1 in initial lymphatics in tissues of the body wall (e.g., skin, breast, oral tissue) as well as internal organs (e.g., lung, colon, rectum, Fallopian tube) (Figure 3). Like in the lymph collectors of the body wall (Figure 2), we found PPFIBP1 expression in lymph collectors of the small intestine (Figure 3H). Of note, blood vessel endothelial cells always appeared to be negative.

**Figure 4:**
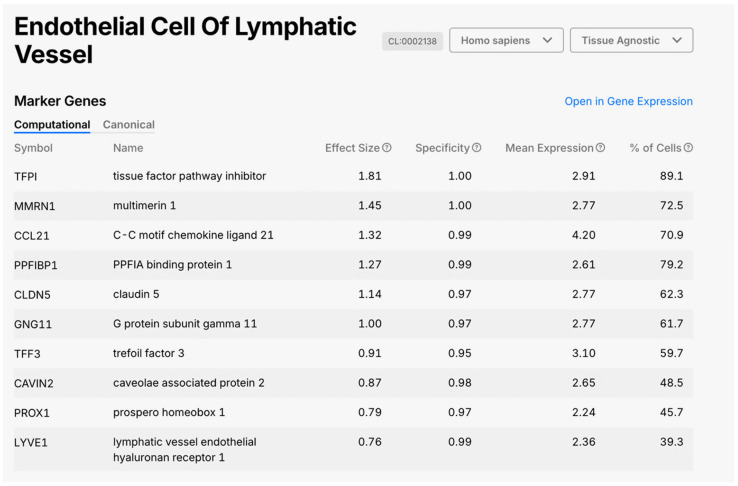
Expression of top 10 LEC marker genes studied by single-cell transcriptomic analysis. PPFIBP1 is listed at position 4. From: (https://cellxgene.cziscience.com/cellguide/CL:0002138; accessed on 17 May 2026).

To further verify expression of PPFIBP1 in LECs, we searched the single-cell data platform CZ CELL × GENE Discover (https://doi.org/10.1101/2023.10.30.563174) for endothelial cells of lymphatic vessels. There, at position four of the marker genes, we found PPFIBP1 even before the commonly used markers PROX1 and LYVE1 (Figure 4).

**Figure 5:**
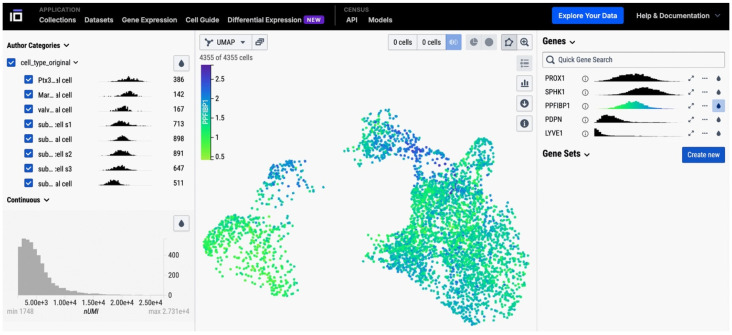
Single-cell RNASeq data analysis of human lymph node LECs. Data shows expression of PPFIBP1 (blue–green) in comparison to other LEC markers. PPFIBP1 is found in all eight LEC subtypes (upper left corner). Expression level of PPFIBP1 is similar to PROX1 and SPHK1, and higher than podoplanin (PDPN) and LYVE1 (upper right corner). Data from [24]. nUMI—number of unique molecular identifiers.

In the Human Protein Atlas, we could not find immunohistochemical evidence for PPFIBP1 expression in lymph node LECs and therefore studied its expression in a single-cell database of human lymph nodes [24], available at: https://cellxgene.cziscience.com/e/cfa3c355-ee77-4fc8-9a00-78e61d23024c.cxg/ (accessed on 17 May 2026). There, we found that PPFIBP1 was expressed in all LEC subtypes, with an expression level comparable to the LEC marker PROX1 and the functionally highly important Sphingosine kinase 1 (SPHK1) (Figure 5). SPHK1 is the rate-limiting enzyme for the production of the lipid mediator sphingosine-1-phosphate (S1P), which is produced by LECs and guides B cells into the efferent lymph of lymph nodes [25].

**Figure 6:**
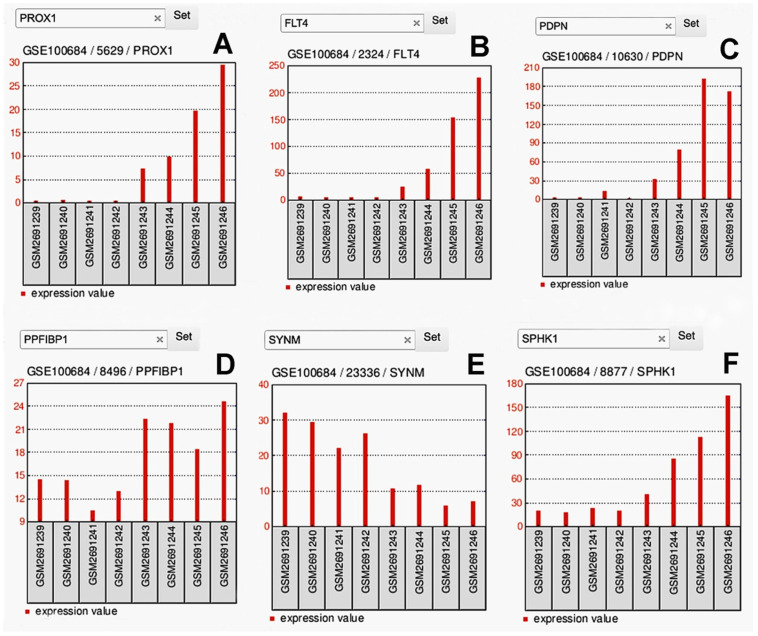
Expression values (Transcripts Per Kilobase Million [TPM], normalized) of four control tissues (left) vs. four Kaposi’s Sarcoma (KS) lesions (right); from GSE100684; see: https://www.ncbi.nlm.nih.gov/geo/geo2r/?acc=GSE100684; accessed on 17 May 2026. Note expression of LEC markers (**A**) PROX1, (**B**) FLT4/VEGFR-3 and (**C**) podoplanin (PDPN) in KS lesions. (**D**) PPFBP1 and (**F**) SPHK1 expression is higher, (**E**) SYNM lower in KS lesions vs. controls.

Kaposi’s Sarcoma (KS) is a mixed-cell tumor of vascular-like cells, spindle cells, immune cells and extravasated erythrocytes, and is based on the infection of a small number of tumor cells with the KS-associated herpesvirus 8 (KSHV). The infected cells have endothelial characteristics and are either CD34-negative (proliferative) or CD34-positive [26]. The KS lesions express LEC markers such as PROX1, FLT4/VEGFR-3 and podoplanin (PDPN). In addition, a clear difference between controls and tumor cells is also found with regard to the expression of PPFIBP1, SYNM and SPHK1 (Figure 6). Thereby, PPFIBP1 and SPHK1 are consistently higher, and SYNM lower in KS lesions vs. control tissue [27].

In summary, at transcriptomic and protein levels we found expression of PPFIBP1 in initial lymphatics, lymph collectors, lymph node sinuses, LECs in vitro and in KS lesions.

### 3.2. Synemin (SYNM; Aka Desmuslin, DMN)

The type-VI intermediate filament (IF) synemin is well known for its interaction with the muscle cell IF desmin (DES) and is typically found in all ‘professional’ contractile cell types [13]. Surprisingly, we observed expression of *SYNM* in foreskin-derived LECs in vitro (Table 1). Expression levels were moderate, most likely due to the fact that IFs are stable proteins with low turnover, and therefore are regularly used in cancer diagnostics. Expression of SYNM in LECs was not regulated by hypoxia (Table 1).

**Figure 7:**
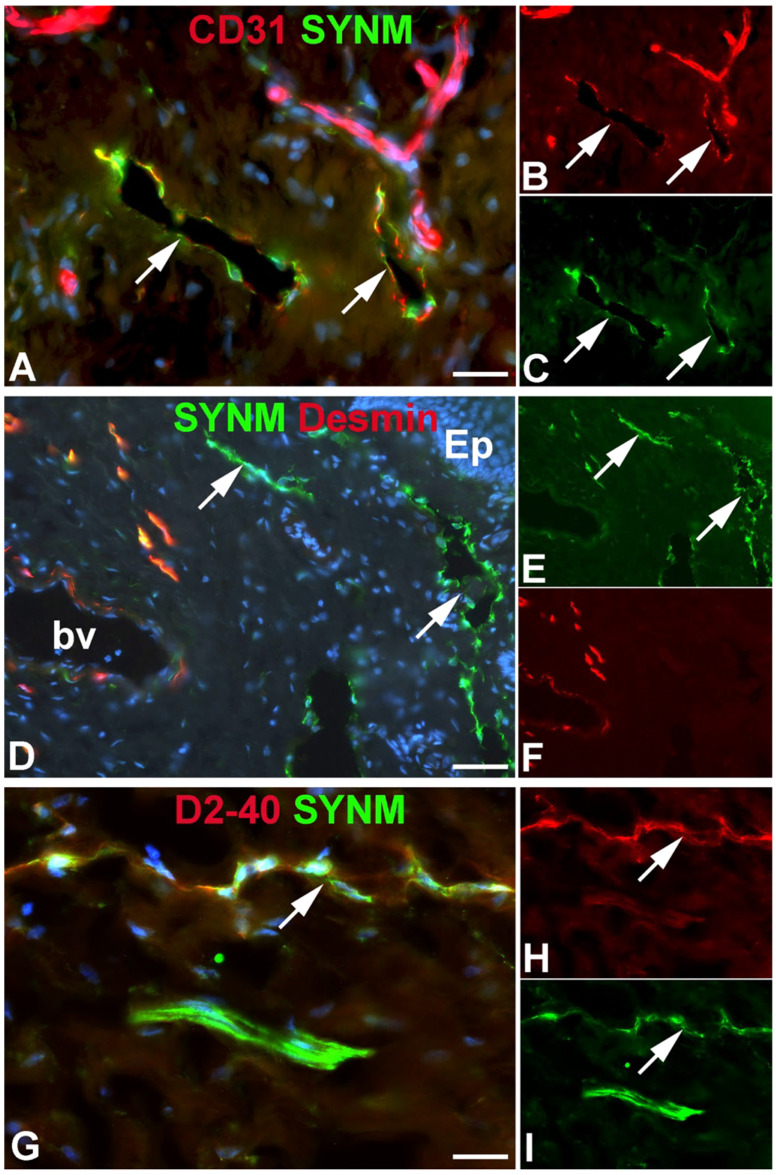
Immunofluorescence (IF) studies on SYNM in human foreskin. Initial lymphatics are marked with arrows. (**A**–**C**) Double IF of anti-SYNM (green) with anti-CD31 (red). Not expression of SYNM in CD31-weak lymphatics. (**D**–**F**) Double IF of anti-SYNM (green) with anti-desmin (red). Desmin stains smooth muscle cells in the dermis, but is not expressed in lymphatics. (**G**–**I**) Double IF of anti-SYNM (green) with anti-podoplanin (D2-40, red). SYNM is expressed in LECs and in smooth muscle cells. (**A**,**D**,**G**) Merged images. Nuclei are counterstained with DAPI (blue). bv—blood vessel; Ep—epidermis. Bar = 25 µm in (**A**,**G**), and 50 µm in (**D**).

We studied SYNM in frozen sections of human foreskin in combination with the endothelial marker CD31/PECAM1, the muscle cell marker desmin, and the LEC marker D2-40/podoplanin. As noted above, CD31 is highly expressed in BECs, while it is found at lower levels in LECs. The CD31-low lymphatics expressed SYNM (Figure 7A–C). The LECs did not express desmin, which was co-expressed with SYNM in dermal smooth muscle cells (Figure 7D–F). Expression of SYNM in LECs was confirmed by double-staining with the LEC marker D2-40/podoplanin (Figure 7G–I).

**Figure 8:**
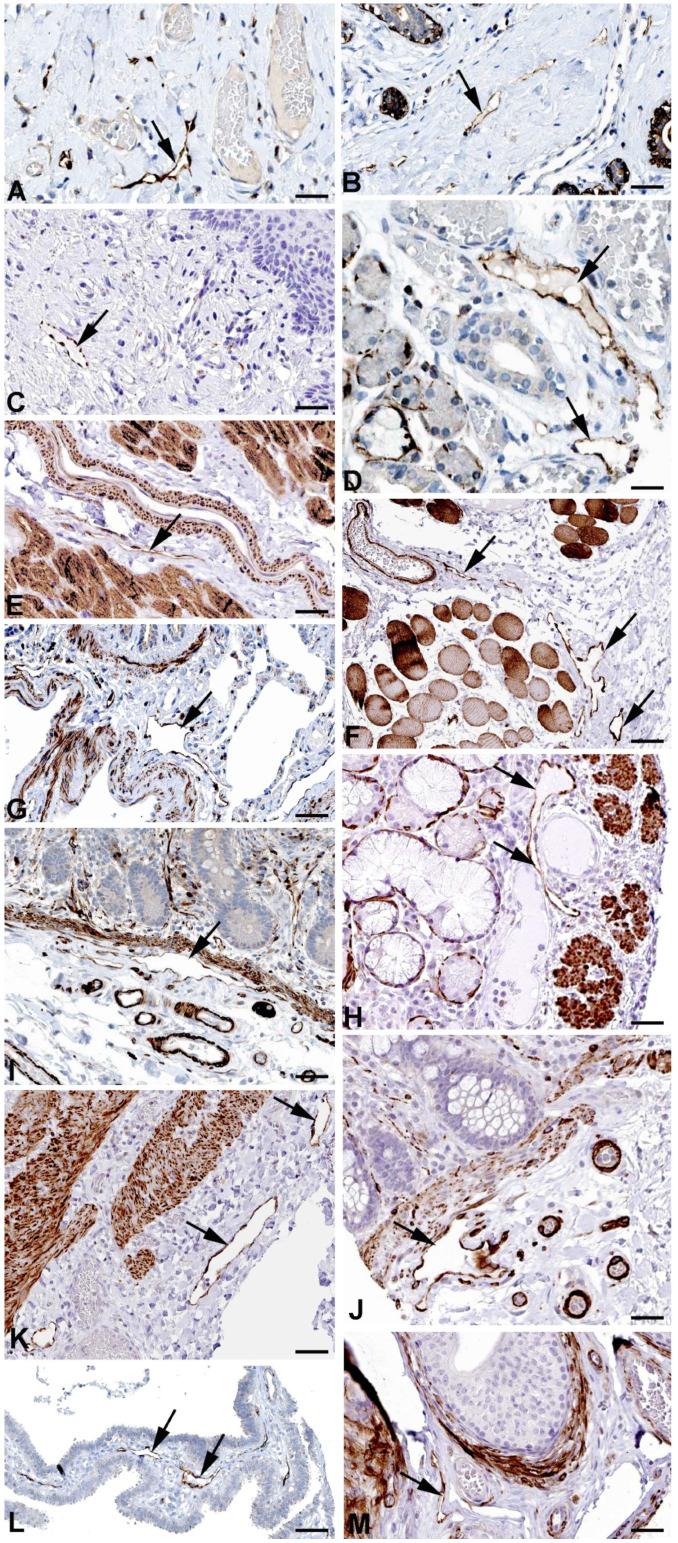
Immunohistochemical studies on SYNM in adult human tissues. Note the staining of initial lymphatics (arrows) of body wall tissues and internal organs, in addition to smooth muscle cells, myoepithelial cells, skeletal muscle fibers and cardiomyocytes. Data from the Human Protein Atlas [22]. (**A**) Skin, patient id 3263, male 69 y; antibody CAB017192; Bar = 25 µm. (**B**) Breast, patient id 2773, female 23 y; antibody CAB017192; Bar = 40 µm. (**C**) Vagina, patient id 4959, female 42 y; antibody HPA044200; Bar = 40 µm. (**D**) Salivary gland, patient id 1845, male 63 y; antibody CAB017192; Bar = 25 µm. (**E**) Heart, patient id 2398, male 62 y; antibody HPA040066; Bar = 40 µm. (**F**) Skeletal muscle, patient id 4905, female 75 y; antibody HPA040066; Bar = 40 µm. (**G**) Lung, patient id 2268, female 49 y; antibody CAB017192; Bar = 40 µm. (**H**) Bronchus, patient id 4552, male 56 y; antibody HPA040066; Bar = 40 µm. (**I**) Colon, patient id 3266, male 73 y; antibody CAB017192; Bar = 40 µm. (**J**) Rectum, patient id 4489, female 58 y; antibody HPA040066; Bar = 40 µm. (**K**) Gallbladder, patient id 3472, male 26 y; antibody HPA040066; Bar = 40 µm. (**L**) Fallopian tube, patient id 2447, female 32 y; antibody CAB017192; Bar = 80 µm. (**M**) Epididymis, patient id 4972, male 25 y; antibody HPA040066; Bar = 40 µm.

**Figure 9:**
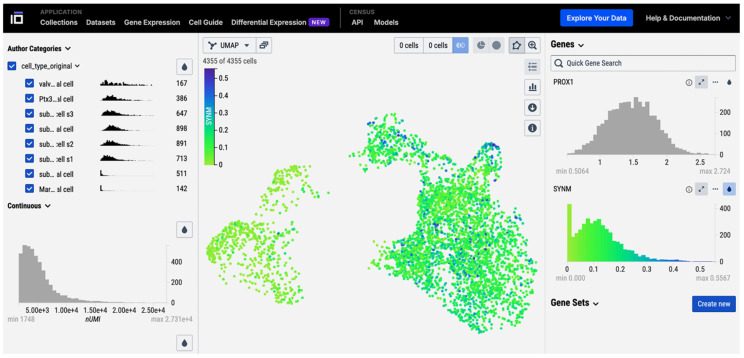
Single-cell RNASeq data analysis of human lymph node LECs. Data shows expression of SYNM (blue–green) in comparison to the LEC marker PROX1. Only a very small number of LECs express SYNM. Data from [24]. nUMI—number of unique molecular identifiers.

Next, we again used the Human Protein Atlas [22] and systematically studied the immunohistochemically stained organs. We found SYNM in LECs of initial lymphatics in tissues of the body wall (skin, breast, skeletal muscle) as well as internal organs (e.g., salivary glands, vagina, heart, lung, gallbladder, colon, rectum, Fallopian tube, epididymis, and others) (Figure 8). The study also identified the known expression domains in typical contractile cells such as myoepithelial cells, smooth muscle cells, cardiomyocytes, and skeletal muscle fibers. We did not find anti-SYNM-positivity in our lymph collector specimens, and could also not find positive lymph collectors in the Human Protein Atlas. Also, the single-cell RNASeq data analysis of human lymph node LECs did not reveal significant expression of SYNM (Figure 9). In Kaposi’s Sarcoma (KS), however, low expression of SYNM could be observed (Figure 6). Expression was constantly lower than in controls. However, it remained unknown what percentage of muscle cells was present in the controls.

In summary, at transcriptomic and protein levels we found expression of SYNM in initial lymphatics. Lymph collectors and lymph node sinuses were mostly negative, whereas KS lesions showed low positivity.

## 4. Discussion

### 4.1. PPFIBP1

PPFIBP1 (aka liprin β1 or PPFIA binding protein 1) was detected in human lymphatics several years ago but has not been intensively studied since then [12]. It was shown that PPFIBP1 is expressed in human small intestinal lacteals together with PROX1. Additionally, in human dermal microvascular endothelial cells, which are a mixture of BECs and LECs, PPFIBP1 was co-expressed in PROX1-positive LECs and absent from PROX1-negative BECs. This already suggested that PPFIBP1 might be a suitable marker for LECs. However, the authors suggested that PPFIBP1 is primarily present in intestinal lymphatic vessels and only to a lesser extent in dermal lymphatic vessels. Our RNASeq analyses revealed high expression of PPFIBP1 in three dermal LEC isolates. Expression was stable under hypoxic conditions. Our IF studies confirm expression of PPFIBP1 in dermal, CD31-weak initial lymphatics, while in dermal blood vessels we could not detect PPFIBP1. Additionally, in human hypodermal thigh lymph collectors the LECs are PPFIBP1-positive, while the vasa vasorum in the tunica media of the collector are PPFIBP1-negative. Our data show that PPFIBP1 is well suited for visualizing dermal lymphatic vessels. This is also confirmed by the immunohistochemical analyses in the Human Protein Atlas. Here, too, images of the lymphatic vessels in the breast, skin, and oral tissue can be observed. The lymphatic vessels of internal organs are also PPFIBP1-positive. This was demonstrated by immunohistochemical analysis of the colon, rectum, lungs and Fallopian tube. In a specimen of the small intestine, we observed PPFIBP1 in a lymph collector, indicating that both initial lymphatics and collectors of the body wall and internal organs are characterized by PPFIBP1 expression. Single-cell RNASeq analyses confirm that PPFIBP1 is among the top-ranking markers of LECs (https://cellxgene.cziscience.com/cellguide/CL:0002138; accessed on 17 May 2026).

Additionally, in lymph nodes which contain LECs with highly specialized functions, PPFIBP1 is consistently expressed. Its expression levels are comparable to those of PROX1 and SPHK1, and significantly higher than those of podoplanin and LYVE1. While PROX1 is a master regulator of LEC development and maintenance [28,29], SPHK1 is the rate-limiting enzyme for the production of the lipid mediator sphingosine-1-phosphate (S1P). S1P is present in all body fluids, but highest in lymph. It is secreted by lymph node LECs and attracts B cells into the efferent lymph for recirculation [25].

We can only tentatively determine whether immunohistochemical analysis of PPFIBP1 can be used in the diagnosis of Kaposi’s Sarcoma. PPFIPB1 expression is higher in the KS group than in the control groups; however, nothing can be said about the exact cellular composition of the controls (https://www.ncbi.nlm.nih.gov/geo/geo2r/?acc=GSE100684; accessed on 21 May 2026). KS contains cells with BEC and LEC characteristics [26]. However, the functions of PPFIBP1 in LECs remain largely unknown. As a scaffold protein it mediates the assembly of target proteins into large complexes adjacent to the cell membrane, thereby regulating numerous cellular activities, especially those that involve cell–matrix interactions at focal adhesions [10]. Dysregulation of cell–matrix interactions is frequently observed in the metastasis of tumor cells. The often pathologically altered expression of PPFIBP1 in tumor cells may be related to this [9,30]. The control of cell–matrix interaction may also be the reason why PPFIBP1 is necessary for maintaining the functionality of lymph vessels. Initial lymphatics are focally connected to the extracellular matrix by anchoring filaments [31], which is important for the function of the microvalves [32]. The knockdown of PPFIBP1 in Xenopus tadpoles induces edema formation [12].

### 4.2. Synemin

In humans, synemin (*SYNM*; aka desmuslin, *DMN*) exists in two major splice variants. However, whether these have different functions is largely unknown [15]. SYNM is well known for its interaction with desmin (DES) [33]. It is therefore a marker of all muscle cell types [13]. Our studies, including those conducted with the Human Protein Atlas, have also shown that SYNM and DES are excellent markers for all muscle cell types, including the myoepithelial cells of many glands. In KS lesions, double-staining of SYNM and DES might differentiate between muscle cells and LECs, the latter being DES-negative but positive for SYNM and LEC markers like D2-40, PROX1 or VEGFR-3.

SYNM also directly interacts with vimentin, which is highly expressed in LECs [1], but expression of SYNM in LECs has not been reported yet. We show that SYNM is moderately and stably expressed at the RNA level in human dermal LECs, and is well detectable in initial lymphatics with immunohistology. Moderate levels of RNA expression are not surprising. Intermediate filaments are stable proteins and systematically used in cancer diagnostics [34]. Since SYNM seems to be specifically present in initial lymphatics, but not in collectors and lymph node sinuses, it might be involved in the functioning of the microvalves, which direct the influx of intercellular fluid into the lymphatics. However, this remains speculative since lymphedema was not reported in *Synm*-knockout mice [35]. But lymphedema is often difficult to diagnose and differentiate from cardiac edema in mice (especially when it is not expected), and molecular differences in human and murine lymphatics are not uncommon.

### 4.3. Limitations

We examined the RNA and protein expression of PPFIBP1 and SYNM in vitro in human LECs derived from the foreskin, using immunofluorescence in frozen sections of juvenile foreskin, as well as in publicly available databases. We have not conducted any studies on adult organs and tissues ourselves and have therefore not performed any double staining with established LEC markers in such tissues. However, the morphology and location of lymphatic vessels are so distinctive that even individual immune-stains of the Human Protein Atlas (HPA) can be used for analysis. Our findings strongly suggest that PPFIBP1 and SYNM have great potential to be used as markers for lymphatic vessels. Nevertheless, a much larger number of studies on normal and pathological tissues and organs, including lymphoid tissues, in combination with established markers, must be conducted to verify the findings. Besides lymphatic malformations, tumor tissues have to be studied. In tumors, endothelial cells often lose their original identity and express molecules that are found in the cells only transiently during embryonic development; e.g., *FLT4*/VEGFR-3 is functionally important in embryonic blood vessels before it becomes largely restricted to the lymphatics [36].

Furthermore, the antibodies used in our studies are polyclonal. The risk of cross-reactivity must always be taken into account. However, the staining pattern of the synemin antibody was entirely as expected, and both antibodies were classified as “approved” in the protein array by the HPA. Nevertheless, the use of monoclonal antibodies should be preferred in clinical diagnostics.

## 5. Conclusions

There is heterogeneity of lymphatic endothelial cells (LECs) in initial lymphatics, collectors and lymph nodes, and the commonly used markers for LECs do not consistently stain LECs in all sections of the lymphatic system. In cancers, blood vessels can express LEC markers such as VEGFR3/*FLT4*, and in lymphatic malformations, heterogeneity of LEC markers has been observed. Additional markers of LECs can help to distinguish between blood vessels and lymphatics. Here, we have presented the expression of two molecules which have received little or no attention in lymphatic research to date: PPFIBP1 and synemin. Both of them appear to be of great value for the characterization of LECs. We are aware that our study is a starting point, and will probably stimulate the validation of monoclonal antibodies for the use of PPFIBP1 and SYNM in the diagnosis of lymphatics in health and disease.

## Figures and Tables

**Table 1:** *PPFIBP1* is highly expressed in human dermal lymphatic endothelial cells (HDLEC-5/6/7) in vitro under normoxia (21% pO_2_) and 1% pO_2_ hypoxia (Hypox-5/6/7). *SYNM* is moderately expressed. The expression of the two genes is not significantly regulated by hypoxia. Read counts are shown.

Gene_Id	Gene_Name	Chromos.	Biotype	HDLEC-5	Hypox-5	HDLEC-6	Hypox-6	HDLEC-7	Hypox-7
ENSG00000110841	PPFIBP1	12	protein-coding	10.059	12.026	21.261	24.781	3.265	3.826
ENSG00000182253	SYNM	15	protein-coding	1.039	855	2.391	4.232	558	380

## Data Availability

Own data are included in the manuscript. Additional immunohistochemical data are found at the Human Protein Atlas (https://www.proteinatlas.org/ accessed on 7 June 2026). In addition to our own analyses, the transcriptome of LECs was studied at https://cellxgene.cziscience.com/cellguide/CL:0002138 accessed on 7 June 2026 and https://cellxgene.cziscience.com/e/cfa3c355-ee77-4fc8-9a00-78e61d23024c.cxg/ accessed on 17 May 2026. The transcriptome of Kaposi’s Sarcoma was studied at: https://www.ncbi.nlm.nih.gov/geo/geo2r/?acc=GSE100684 accessed on 17 May 2026.
